# Exploring Associations between C-Reactive Protein and Self-Reported Interoception in Major Depressive Disorder: A Bayesian Analysis

**DOI:** 10.3390/brainsci13020353

**Published:** 2023-02-18

**Authors:** Michael Eggart, Juan Valdés-Stauber, Bruno Müller-Oerlinghausen, Martin Heinze

**Affiliations:** 1 Brandenburg Medical School Theodor Fontane, Faculty of Health Sciences, 16816 Neuruppin, Germany; 2 Department of Psychiatry and Psychotherapy I, Ulm University and Center for Psychiatry Südwürttemberg, 88214 Ravensburg, Germany; 3 Faculty Social Work, Health and Nursing, Ravensburg-Weingarten University of Applied Sciences, 88250 Weingarten, Germany; 4 Brandenburg Medical School Theodor Fontane, Faculty of Medicine and Psychology, 16816 Neuruppin, Germany; 5 Charité—Universitätsmedizin Berlin, 10117 Berlin, Germany; 6 Department of Psychiatry and Psychotherapy, Brandenburg Medical School Theodor Fontane, Immanuel Klinik Rüdersdorf, 15562 Rüdersdorf, Germany

**Keywords:** major depressive disorder, interoception, inflammation, C-reactive protein, CRP, fatigue, sickness behavior, protective effect, psychoneuroimmunology, Bayesian inference

## Abstract

Major depressive disorder (MDD) is associated with dysfunctional self-reported interoception (i.e., abnormal perception of the body’s physiological state) and systemic inflammation, both of which adversely affect treatment response. In this study, we explored associations between C-reactive protein (CRP) and self-reported interoception, to gain more insight into the pathophysiology of interoceptive impairments in MDD. We also aimed to replicate previous findings on the associations of depression and fatigue severity with CRP. The study included 97 depressed individuals, who completed self-administered questionnaires (Multidimensional Assessment of Interoceptive Awareness (MAIA-2); Beck Depression Inventory-II, Multidimensional Fatigue Inventory). CRP concentrations were analyzed in the serum using a particle-enhanced turbidimetric immunoassay. We applied Bayesian inference to estimate robust effect parameters from posterior distributions based on MCMC sampling, and computed Bayes factors (BF_10_) as indices of relative evidence. The bivariate analysis supported evidence against associations between CRP and self-reported interoception (BF_10_ ≤ 0.32), except for one dimension (Not-Distracting: r = 0.11, BF_10_ > 0.43, absence of evidence). Positive correlations with overall depression (r = 0.21, BF_10_ = 3.19), physical fatigue (r = 0.28, BF_10_ = 20.64), and reduced activity (r = 0.22, BF_10_ = 4.67) were found. The multivariate analysis showed moderate evidence that low-grade inflammation predicted higher scores on the MAIA-2 Not-Worrying scale (β = 0.28, BF_10_ = 3.97), after controlling for relevant confounders. Inflammatory responses, as measured by CRP, may not be involved in the pathophysiology of dysfunctional self-reported interoception. However, systemic low-grade inflammation could potentially exert a protective effect against worries about pain or discomfort sensations. An immunological involvement in interoceptive impairments cannot be ruled out until future studies considering additional biomarkers of inflammation replicate our findings.

## 1. Introduction

Major depressive disorder (MDD) is a common mental disorder affecting mood, cognition, psychomotor activity, behavior, and bodily self-awareness [1,2,3]. Patients often suffer from painful and non-painful somatic symptoms, such as fatigue, that increase the risk of recurrence, chronicity, and suicidality [4]. MDD can lead to significant disability, reduced quality of life, and increased risk of somatic morbidity/mortality [3].

The pathogenesis of MDD is poorly understood. The hypothesis that serotonin deficiency is causally related to depression has repeatedly been put forward, although the available evidence does not suggest any association [5]. In addition, a number of inconsistent research findings have highlighted neurobiological differences between depressed individuals and healthy controls [6], leading to a mistaken conceptualization of depression as a “brain disorder” [7]. Cumulative evidence suggests that dysregulated inflammatory responses are involved in the pathophysiology of MDD across a broad spectrum of pro-inflammatory markers (for a review, see [8]). For example, levels of peripheral C-reactive protein (CRP)—an acute phase reactant that is synthesized by hepatocytes in response to pro-inflammatory cytokines (in particular interleukin-6 (IL-6))—are associated with overall depression severity [8,9]. However, the findings are mixed, after adjustment for body mass index (BMI), which typically attenuates the association [9]. A meta-analysis estimated that approximately one quarter of patients exhibit low-grade inflammation, defined as CRP > 3.0 mg/L [10]. Notably, elevated serum CRP is positively related to the somatic symptom profile of atypical depression [11]: This “inflammatory phenotype” includes symptoms of fatigue, hypersomnia, leaden paralysis, increased appetite, weight gain, and anhedonia [12,13,14]. Recent research found higher CRP levels in treatment-resistant patients compared with treatment responders [15], which is consistent with a longitudinal study identifying baseline CRP as a predictor of antidepressant response [16]. It has been speculated that inflammatory processes could explain the high comorbidity of cardiovascular disease, diabetes mellitus, schizophrenia, and MDD [17]. However, there is an ongoing debate regarding (a) the causal involvement of chronic low-grade inflammation in MDD pathogenesis, suggesting a potential bidirectional relationship; (b) the factors contributing to central/peripheral immune system dysregulation; and (c) the appropriate immunological target for deriving new antidepressant treatments [8]. Overall, a physiological state of chronic low-grade inflammation is prevalent in MDD, which is associated with an energy-related/somatic symptom profile that adversely affects prognosis.

Interoception is the sense of the physiological condition of the body [18], including the interpretation and integration of signals arising from within the body [19]. Primarily, interoception shapes the afferent part of homeostasis, which affects urges, somatic feelings, and adaptive behaviors (e.g., glucose level ↓ → hunger → search for food → eating) [19]. Moreover, a growing body of research has shown that interoception is a central component of emotional experience and cognition [20], which is consistent with early theories of emotion [21]. Dysfunctional interoception (i.e., a maladaptive response to somatic signals) has been identified as a transdiagnostic correlate of mental disorders, e.g., in anxiety, addiction, eating, somatic symptoms, and mood disorders [19]. For example, cumulative evidence suggests that MDD is associated with a blunted heartbeat perception accuracy, which is related to decision-making difficulties and low affect intensity [22]. In the present study, we focus on self-reported interoception, another dimension of interoception, defined as the individual disposition to be focused on subjective interoceptive states by differentiating between (dys)functional attention styles and regulatory aspects [23,24]. A recent study assessed self-reported interoception in individuals suffering from MDD using an established scale, the Multidimensional Assessment of Interoceptive Awareness (MAIA), and found a lack of confidence in body sensations [25]. In addition, changes on several MAIA scales were identified as mediators of antidepressant treatment effects [26,27] and independently predicted treatment response in hospitalized patients [28]. In addition, thresholds for the minimum important difference were established for the MAIA, to classify patients who achieved remission after depression treatment [29]. Therefore, impaired interoceptive functioning is increasingly recognized as a target for the treatment of mood disorders [30].

The pathophysiology of dysfunctional self-reported interoception in MDD is unclear and requires further clarification [19]. Interoception research has historically focused on neural mechanisms rather than immunological processes [31], although acute infections and MDD share phenomenological commonalities in their clinical presentation (e.g., anhedonia, fatigue, loss of appetite, insomnia, social withdrawal); a symptom complex that has been termed “sickness behavior” [8]. A recent study suggested that levels of peripheral blood inflammatory biomarkers (CRP, IL-6, and neutrophils) are associated with decreased functional connectivity in the brain networks that are involved in the processing of interoceptive signals [32]. However, to the best of our knowledge, a potential association between pro-inflammatory states and dysfunctional self-reported interoception has never been explored in MDD. It is therefore an open question whether dysfunctional self-reported interoception can be regarded as another component of sickness behavior.

In this study, we explored associations between serum CRP and multidimensional self-reported interoception in hospitalized patients suffering from MDD. Due to recent reports of low replicability of research findings in the clinical disciplines, we referred to the Bayesian framework to increase the credibility of our analysis [33,34]. First, we studied bivariate correlations between serum concentrations of CRP and facets of self-reported interoception. Second, we investigated whether self-reported interoception is predicted by CRP, after adjusting for relevant confounders. We sought to substantiate the validity of our results by replicating previously presented evidence of a positive association between CRP levels and severity of depression and body-related experience of fatigue [8,9,12,13,14].

## 2. Materials and Methods

The ethics committee of Ulm University approved this study (registration number: 13/17). The principles of the Declaration of Helsinki were met, and written informed consent was obtained from the recruited patients.

### 2.1. Procedure

This study involved a secondary analysis of data that were gathered in a longitudinal, naturalistic trial investigating the effects of self-reported interoception on treatment outcomes in hospitalized patients suffering from MDD. Details on the procedure of participant recruitment, inclusion/exclusion criteria, and a study flow chart are reported in the companion paper [28] and briefly summarized here: study participants were included if they were at least 18 years old and had a main diagnosis of MDD, which was assessed by trained psychiatric specialists according to ICD-10 criteria [35]. Patients were excluded if they had a diagnosis of psychosis, drug addiction, intellectual disability, or no knowledge of German. Study data were collected within 48 h after hospital admission.

### 2.2. Participants

We performed a cross-sectional analysis by including patients from the baseline sample (*N* = 110) who were consecutively admitted to a psychiatric hospital ward that is specialized for the treatment of depression (Department of Psychiatry and Psychotherapy I, Ulm University, Center for Psychiatry Südwürttemberg, Weißenau, Germany). CRP levels were available in blood samples from 98 patients. One patient was excluded from the analysis because of a high CRP concentration (90.5 mg/L; polytrauma after suicide attempt), potentially biasing statistical analyses. Therefore, the study included 97 patients, of whom 37.11% experienced suicidal ideation, which is representative of inpatient MDD samples [36].

### 2.3. Measures

#### 2.3.1. Multidimensional Assessment of Interoceptive Awareness, Version 2 (MAIA-2)

The MAIA-2 assesses self-reported interoception on a 6-point Likert scale based on 37 items. The instrument includes eight dimensions, internal consistency reliabilities (McDonald’s *ω*) for the present study are reported in brackets [29]: (1.) Noticing (“awareness of uncomfortable, comfortable, and neutral body sensations”; *ω* = 0.64), (2.) Not-Distracting (“tendency not to ignore or distract oneself from sensations of pain or discomfort”; *ω* = 0.68), (3.) Not-Worrying (“tendency not to worry or experience emotional distress with sensations of pain or discomfort”; *ω* = 0.68), (4.) Attention Regulation (“ability to sustain and control attention to body sensations”; *ω* = 0.84), (5.) Emotional Awareness (“awareness of the connection between body sensations and emotional states”; *ω* = 0.87), (6.) Self-Regulation (“ability to regulate distress by attention to body sensations”; *ω* = 0.74), (7.) Body Listening (“active listening to the body for insight”; *ω* = 0.76), (8.) Trusting (“experience of one’s body as safe and trustworthy”; *ω* = 0.86). The MAIA-2 has been validated for clinically depressed samples, showing adequate psychometric properties and the ability to discriminate between treatment response groups [29]. Low scores on its eight dimensions are indicative of dysfunctional self-reported interoception.

#### 2.3.2. Beck Depression Inventory-II (BDI-II)

The BDI-II is a self-rating scale assessing the severity of depression based on 21 different symptoms, which are rated on a 4-point Likert scale. This instrument demonstrated appropriate reliability and validity in previous research [37]. We found good internal consistency and reliability in the present study (*ω* = 0.89).

#### 2.3.3. Multidimensional Fatigue Inventory (MFI-20)

The MFI-20 has been validated in the general population and in clinical samples, to assess five facets of fatigue in 20 items, which are rated on a 5-point Likert scale [38]. Internal consistency reliabilities for the present study are reported in brackets: (1.) general fatigue (*ω* = 0.67), (2.) physical fatigue (*ω* = 0.80), (3.) mental fatigue (*ω* = 0.74), (4.) reduced activity (*ω* = 0.80), (5.) reduced motivation (*ω* = 0.67). Previous research reported adequate psychometric properties for the MFI-20 [38].

### 2.4. C-Reactive Protein (CRP)

Blood samples were drawn from the antecubital vein the morning after admission at 7:30–8:30 a.m. using S-Monovette^®^ serum tubes (Sarstedt, Nümbrecht, Germany). CRP was analyzed in the Laboratory of the Department of Psychiatry and Psychotherapy I (Center for Psychiatry Südwürttemberg, Weißenau, Germany) on a Dimension^®^ Clinical Chemistry System (Siemens Healthcare Diagnostics Inc., Erlangen, Germany) using a particle-enhanced turbidimetric immunoassay (Dimension^®^ Flex^®^ reagent cartridge CRP, Siemens Healthcare Diagnostics Inc., Erlangen, München), which yields a minimum sensitivity of 0.5 mg/L. The intra-assay coefficient of variation was 3.80%, the inter-assay coefficient of variation was 4.30%. We referred to established CRP cut-points following recommendations of the American Heart Association [39]: <1.0 mg/L; 1.0–3.0 mg/L; 3.1–10.0 mg/L; >10.0 mg/L. These groups have been widely applied in depression research: 3.0 < CRP ≤ 10.0 mg/L has been defined as indicative of low-grade inflammation, and CRP > 10.0 as indicative of acute inflammation [10]. With reference to recently published recommendations, the analyses included patients with CRP > 10.0 mg/L, to obtain more consistent model estimates, followed by a sensitivity analysis excluding these cases [40]. Due to significant deviations from normality, CRP levels were log-normalized (log_10_ CRP) according to established procedures [9] (Shapiro–Wilk test pre-transformation: *W* = 0.69, *p* < 0.01; post-transformation: *W* = 0.98, *p* = 0.21; findings were confirmed using Q–Q plots (not reported)).

### 2.5. The Bayesian Framework

The mathematical fundamentals of Bayesian inference have been described elsewhere (e.g., [41]). The Bayesian framework has several advantages over the frequentist (i.e., null hypothesis significance testing) approach, which will be briefly discussed [42,43]: First, Bayesian analysis can be an alternative to overcome reliance on *p*-values, which have come under increasing criticism [44]. For example, non-significant results (i.e., *p* > 0.05) may be misinterpreted as evidence for the absence of an effect. However, absence of statistical evidence is not evidence of absence [45]. The Bayesian framework allows quantifying support *for* the null hypothesis (*H*_0_) and not solely against it. Second, rather than computing fixed effect sizes (e.g., a point estimate of the bivariate population correlation), Bayesian inference estimates the probability density of any parameter that is described by the posterior distribution. Robust measures of centrality (e.g., the median) and uncertainty (e.g., credible intervals) can be derived from the posterior distribution, as well as indices of significance (see below). The posterior distribution is based on a prior (i.e., the prior probability distribution), which is updated using observed data according to Bayes’ theorem. Hence, the posterior distributions of the present study may be used as priors for future research. Third, the Bayesian framework provides methods for gaining more accurate parameter estimates out of noisy data or small sample sizes, by achieving better type I error control. These characteristics may be important for improving the reproducibility of research [46]. Finally, Bayesian statistics are flexible for model comparisons and the results are more intuitive to interpret, as opposed to indices from the frequentist approach (e.g., credible intervals refer to a specific probability that an effect lies within an interval, whereas this is not true for the frequentist confidence interval). However, one disadvantage of Bayesian inference is the computational cost, which escalates as the number of model variables increases.

In the present study, we used the following Bayesian indices: the robust median of the posterior distribution (measure of centrality), the 95% highest density interval (95% HDI) as the credible interval (measure of uncertainty), and the Bayes factor (BF, measure of effect significance) as an index of relative evidence that enables decisions about rival models, i.e., the alternative (*H*_1_) vs. null hypothesis (*H*_0_) given the observed data (*D*). The BF is conceptualized as the updating factor of the prior odds *p(H*_1_*)/p(H*_0_*)* and defines the ratio of the marginal likelihoods: BF_10_ = *p(D|H*_1_*)/p(D|H*_0_*)*. For example, BF_10_ = 2 means that, based on the observed data, *H*_1_ is twice as likely as H_0_, whereas BF_10_ = 0.50 means that *H*_0_ is twice as likely as *H*_1_. According to widely accepted classification thresholds [47], a BF_10_ in the range of 1–3 (^1^/_3_–1) was considered as anecdotal, 3–10 (^1^/_10_–^1^/_3_) as moderate, 10–30 (^1^/_30_–^1^/_10_) as strong, 30–100 (^1^/_100_–^1^/_30_) as very strong, and >100 (<^1^/_100_) as indicative of extreme evidence for *H*_1_ (for *H*_0_, respectively). A BF_10_ = 1 suggests absence of statistical evidence. It has to be noted that BF thresholds represent rough orientation points, as opposed to the dichotomized nature of *p*-values (*p* < 0.05).

### 2.6. Data Analysis

The statistical analyses were computed in R 4.1.2 [48]. To investigate bivariate associations, Bayesian correlation tests were performed using the ‘correlationBF’ function from the R package BayesFactor [49]. BFs were computed using the Savage–Dickey density ratio [50]. The posterior distributions were summarized with the ‘describe_posterior’ function from the R package bayestestR [51]. Mathematical details on the Bayesian correlation test have been described by Ly et al. [52]. We used the default priors of the BayesFactor package to run the analyses, by referring to a shifted beta distribution and a scaling factor of $\gamma_{1}=1/3$. Posterior distributions were estimated by following the Markov chain Monte Carlo (MCMC) method with 10,000 iterations. Considering previous research that showed positive correlations with CRP, one-sided BFs were calculated for the BDI-II and MFI-20 scales (*H*_0_: *ρ* = 0; *H_1A_*: *ρ* > 0), whereas two-sided BFs were calculated for the correlations between CRP and the MAIA-2 scales, due to limited prior information about the direction of the effect (*H*_0_: *ρ* = 0; *H_1B_*: *ρ* ≠ 0). In a sensitivity analysis, we examined the impact of several prior distributions with differing scaling factors ($\gamma_{2}=1/\sqrt{27}$; $\gamma_{3}=1/\sqrt{3}$; $\gamma_{4}=1$) on the stability of BFs, to investigate the robustness of our findings [53].

In a Bayesian generalized linear multiple regression analysis, we investigated the effects of log-normalized CRP on MAIA-2, BDI-II, and MFI-20, by estimating robust median standardized (*β_Median_*) and unstandardized (*b_Median_*) regression coefficients from draws of the posterior distribution. The slopes were adjusted for age, sex, BMI, school education, employment status, somatic comorbidity, depression severity, intake of antihypertensives, and statins, according to previous recommendations [54]. We decided to adjust for somatic comorbidity rather than exclusively for inflammatory/autoimmune diseases, because the number of affected patients was small, limiting the statistical power. In examining the effects of CRP on multidimensional fatigue, we followed previous protocols and excluded depression severity as a covariate, to ensure the comparability of results [9]. A sensitivity analysis was performed by excluding cases with acute inflammation, to further investigate the generalizability of the findings to low-grade inflammation. We used the R package rstanarm [55] for multivariate analysis and referred to the default adjusted priors (regression slopes: normal prior centered at 0.00; intercept: normal prior; residual standard error: exponential prior), which are weakly informative and appropriate for a wide range of analytical situations [55]. For Bayesian parameter estimation, we used a MCMC sampling algorithm with 10,000 iterations, based on four chains. We performed sampling quality checks with numerical and graphical diagnostics: chain convergence was assessed with the potential scale reduction factor $\hat{R}$, which should be close to 1.00 ($\hat{R}$ < 1.10), and inspected with trace plots for each regression coefficient; autocorrelation within a chain was checked by referring to the effective sample size (ESS), which is indicative of sufficient sampling quality for ESS > 1000 [56]; model fit was visually evaluated with posterior predictive checking, i.e., comparing simulated data from the fitted model to the observed data [56]. The BFs as measures of effect significance for regression slopes were computed within a BF top-down analysis using the ‘generalTestBF’ function from the R package BayesFactor [49].

## 3. Results

### 3.1. Participant Characteristics

Sociodemographic and clinical characteristics for the total sample and separated by CRP cut-points are shown in Table 1. The median concentration of CRP was 2.50 mg/L (IQR: 1.50–4.30; range: 0.20–23.70). The CRP distribution was highly skewed, which is also mirrored in the arithmetic mean, *M* = 3.81 (*SD* = 4.14). Twenty-eight patients (28.87%) presented with systemic low-grade inflammation (3.0 < CRP ≤ 10.0 mg/L), and acute inflammation (CRP > 10.0 mg/L) was found in eight patients (8.25%). The arithmetic mean of the transformed log_10_ CRP values was *M* = 0.41 (*SD* = 0.38; IQR: 0.18–0.63; range: −0.70–1.37).

Patients with somatic comorbidity had significantly higher log_10_ CRP levels, Δ*M_Median_* = 0.22 [95% HDI 0.07, 0.38], BF_10_ = 13.00 (two-sided). No differences in CRP concentration were found between women and men, Δ*M_Median_* = −0.05 [95% HDI −0.20, 0.09], BF_10_ = 0.27, or between recurrent and first episode depression, Δ*M_Median_* = 0.06 [95% HDI −0.09, 0.23], BF_10_ = 0.34 (two-sided), respectively. There was extreme evidence for a significant positive correlation between body mass index (BMI) and log_10_ CRP, *r_Median_* = 0.45 [95% HDI 0.28, 0.59], BF_10_ > 1000 (two-sided). Moreover, there was moderate evidence for an absence of (i.e., null) correlation between participant’s age and log_10_ CRP levels, *r_Median_* = −0.01 [95% HDI −0.20, 0.18], BF_10_ = 0.23 (two-sided). CRP was not associated with school (BF_10_ = 0.32) or vocational education (BF_10_ = 0.18).

### 3.2. Zero-Order Correlations between CRP and Self-Rating Scales

**Figure 1:**
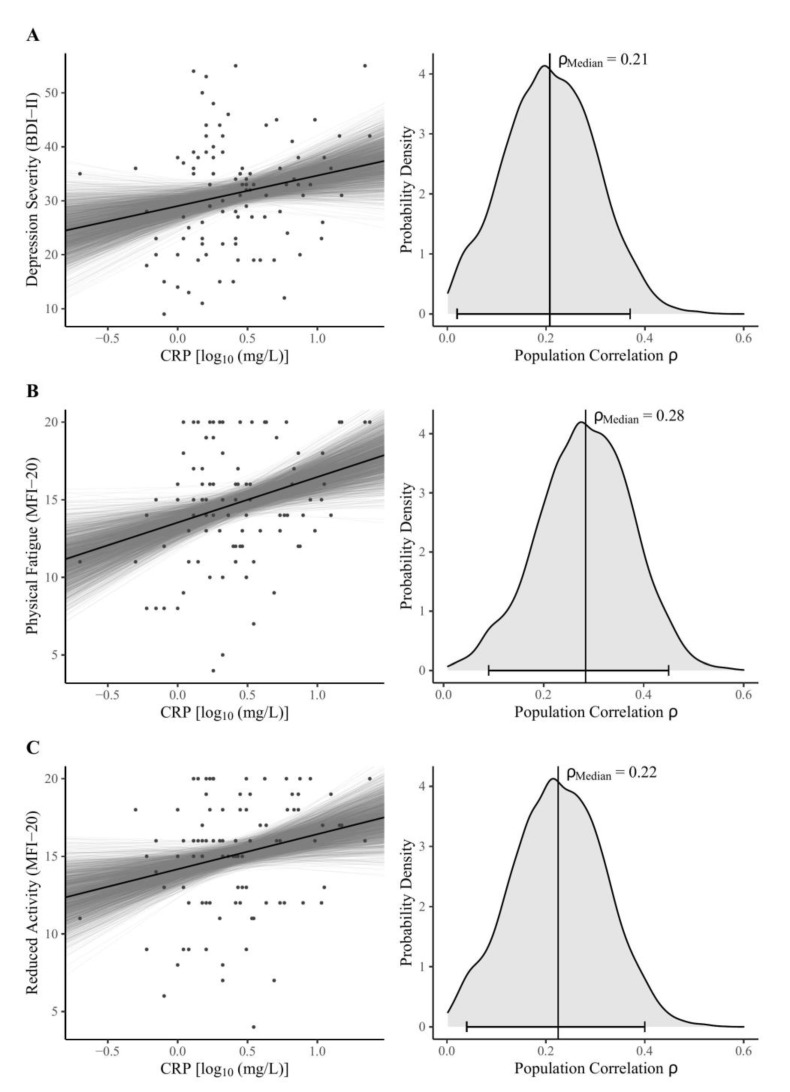
Bayesian model estimates (left) and posterior distributions (right) for the zero-order correlations between log-normalized CRP concentrations and (**A**) BDI-II sum score, the MFI-20 dimensions (**B**) physical fatigue, and (**C**) reduced activity. Note: The left panel shows Bayesian regression lines from 2000 posterior draws (grey lines), scatter plots, and the robust median regression (black line) of the fitted model (BDI-II sum score: unstandardized regression coefficient *b* = 5.62, intercept *ic* = 29.02, *R^2^* = 0.04; MFI-20 Physical Fatigue: *b* = 2.94, *ic* = 13.53, *R^2^* = 0.08; MFI-20 Reduced Activity: *b* = 2.26, *ic* = 14.17, *R^2^* = 0.05). In the right panel, vertical lines indicate the median, as the measure of centrality; the horizontal bottom lines with whiskers show the 95% Highest Density Interval. The probability density distributions were estimated from 4000 posterior draws. Abbreviations: BDI-II = Beck Depression Inventory-II; MFI-20 = Multidimensional Fatigue Inventory; CRP = C-reactive protein; *ρ*_Median_ = estimate of population correlation.

The main findings of the Bayesian correlation analyses are shown in Table 2. Considering the MAIA-2 scales, our data support moderate evidence for *H*_0_, assuming null correlations with peripheral CRP except for the Not-Distracting scale, suggesting no clear conclusions based on our data (absence of evidence). There was moderate evidence for a positive correlation between log-normalized CRP and both depression severity (Figure 1A) and reduced activity (Figure 1B). We found strong evidence for a positive correlation between CRP and physical fatigue (Figure 1C).

The sensitivity analysis (Table 2) showed consistent BFs and qualitatively similar results over a broad range of changing priors. Effect directions were stable, which was also the case under the uniform prior (*γ*_4_ = 1), representing an extreme assumption. These findings support the robustness of the bivariate analysis.

### 3.3. Adjusted Associations between CRP and Self-Rating Scales 

We estimated the effects of log-normalized CRP on multidimensional self-reported interoception, fatigue, and overall depression severity, after adjusting for relevant covariates (Table 3). In a sensitivity analysis, we repeated the computations after exclusion of patients with acute inflammation (CRP > 10.0 mg/L). The multivariate models were not affected by autocorrelation (ESS > 1000), showed chain convergence for all predictors ($\hat{R}$ = 1.00), and an adequate model fit.

**Figure 2:**
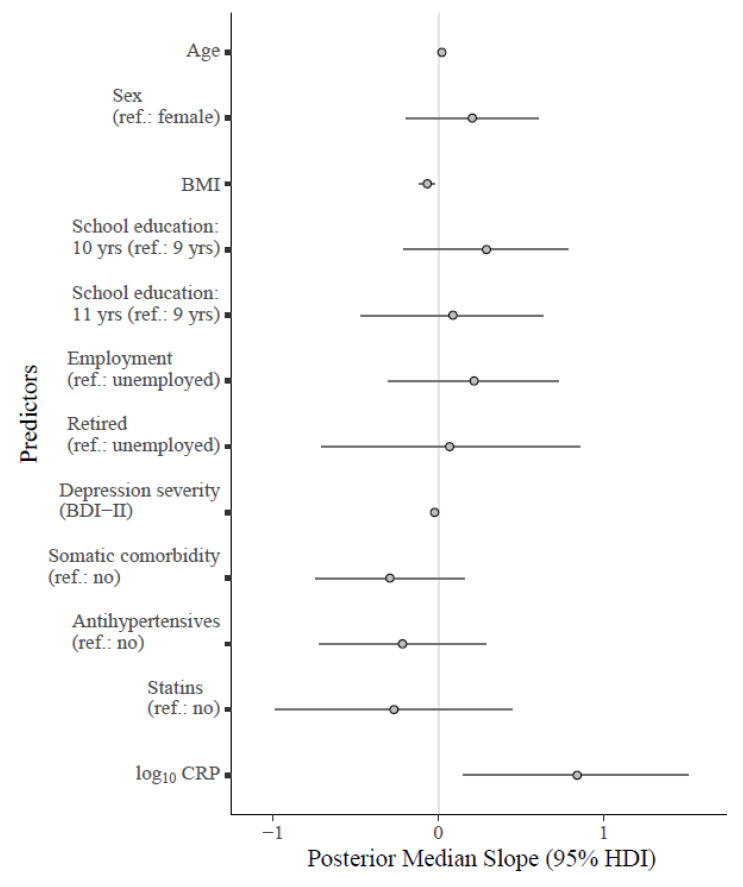
Forest plot for the fitted Bayesian multivariate model regressing the MAIA-2 Not-Worrying scale on log_10_ CRP and relevant covariates. Note: Unstandardized regression coefficients are shown in the plot. The points on the grey lines indicate the median slope derived from the posterior distribution, along with horizontal error bars indicating the 95% HDI. Median standardized slopes *β* [95% HDI] for each predictor: age (0.24 [−0.02, 0.49], BF_10_ = 1.67); sex (0.11 [−0.11, 0.32], BF_10_ = 0.36); BMI (−0.35 [−0.59, −0.11], BF_10_ = 12.86); school education (10 yrs: 0.16 [−0.12, 0.42], 11 yrs: 0.05 [−0.23, 0.33]; BF_10_ = 0.23); employment status (employed: 0.11 [−0.15, 0.36], retired: 0.02 [−0.23, 0.28]; BF_10_ = 0.23); depression severity (−0.26 [−0.50, −0.04], BF_10_ = 4.44); somatic comorbidity (−0.14 [−0.35, 0.09], BF_10_ = 0.53); antihypertensives (−0.10 [−0.33, 0.14], BF_10_ = 0.35); statins (−0.08 [−0.31, 0.14], BF_10_ = 0.44). Cases with acute inflammation were excluded (CRP > 10.00 mg/L). Abbreviations: BDI-II = Beck Depression Inventory-II; BMI = body mass index; 95% HDI = 95% high density interval; log_10_ CRP = log-normalized C-reactive protein (CRP); yrs = years.

The main findings from the bivariate analyses regarding self-reported interoception could be replicated, except for the Not-Worrying scale. The sensitivity analysis identified low-grade inflammation as a predictor of higher scores on the Not-Worrying scale (Figure 2). These results suggest that effects of third variables are involved that were not controlled for in the bivariate analysis and likely obscured the association. In a post-hoc analysis, we screened for potential influential covariates. The significant effect of low-grade inflammation on Not-Worrying subsided after excluding BMI from the analysis (*β_Median_* = 0.19 [95% HDI −0.04, 0.42], *b_Median_* = 0.55 [95% HDI −0.12, 1.24], BF = 1.12) but remained after omitting other covariates. Therefore, we conducted another sensitivity analysis for the Not-Worrying scale by excluding obese patients (BMI ≥ 30.00), to rule out influential effects of extreme values: the regression coefficients were consistent for both overall inflammation (*β_Median_* = 0.35 [95% HDI 0.09, 0.60], *b_Median_* = 1.03 [95% HDI 0.27, 1.80], BF = 2.69) and low-grade inflammation (*β_Median_* = 0.37 [95% HDI 0.11, 0.63], *b_Median_* = 1.14 [95% HDI 0.35, 1.94], BF = 3.97). Evidence against an inflammatory involvement in self-reported interoception (except for Not-Worrying) was demonstrated for the Attention Regulation, Emotional Awareness, Self-Regulation, Body Listening, and Trusting scales. These findings were also consistent after excluding subjects with acute inflammation.

There was moderate evidence that both physical fatigue and reduced activity were positively associated with CRP (Table 3). However, the sensitivity analysis showed that only reduced activity was significantly predicted by low-grade inflammation (moderate evidence), whereas anecdotal evidence indicated against an association between log_10_ CRP and physical fatigue. There was further evidence against an association between log_10_ CRP and general fatigue. The analysis did not support reliable conclusions about the associations with mental fatigue, reduced motivation, and overall depression severity, suggesting absence of evidence.

## 4. Discussions

In the present study, we found preliminary evidence that systemic inflammation measured by peripheral CRP is not associated with dysfunctional self-reported interoception. The validity of our analysis was substantiated by replicating previous findings that showed associations between CRP and depression/fatigue severity [9,12,57]. These results are relevant because abnormal interoception is a core characteristic of depression [29,58,59], with a significant impact on its treatment [26,27,28].

The evidence found against an effect of pro-inflammatory activation on dysfunctional self-reported interoception was contrary to our expectations, for several reasons. First, functional brain imaging findings have suggested an involvement of the insula, a primary region for interoceptive processing [18], in the processing of inflammatory states [32]. Second, it has been shown that peripheral CRP is associated with a somatic symptom and energy-related phenotype of atypical depression [11], which in turn is associated with dysfunctional self-reported interoception [28]. Third, sickness behavior in response to inflammation is, inter alia, mediated by the vagus nerve, which is part of the interoceptive nervous system [8,58]. However, our findings can also be discussed in the light of the active inference theory of interoception [60]. Predictive coding models of MDD suggest an insensitivity to somatic signals, due to noisy afferent inputs and unresolved prediction errors (conceptualized as mismatches between top-down predictions and bottom-up sensations), leading to a “locked in” state of the brain [60]. The resulting sense of disturbed embodiment has also been reported in phenomenological psychopathology, including a ‘corporealization’ of the lived body, i.e., the detachment from vibrant bodily feelings [1]. Abnormal awareness of somatic signals in MDD has also been reported for a subgroup of patients showing reduced heartbeat perception accuracy [22]. Therefore, the insensitivity to vague somatic signals in MDD could lead to a potential uncoupling of bodily feelings from interoceptive signaling of inflammation at the level of consciousness. Given that inflammatory states (a) involve interoceptive pathways [31], (b) are linked to the somatic symptom profile of atypical depression (e.g., leaden paralysis, hypersomnia) [14], but (c) are uncorrelated with dysfunctional self-reported interoception (as shown here for CRP), our findings contribute to a growing body of research that conceptualizes interoception as independent across submodalities at the perceptual level [24,61]. Therefore, evidence against an involvement of CRP in the pathophysiology of dysfunctional self-reported interoception may not be generalized to other dimensions of interoception. Future research should further clarify the cause of maladaptive self-reported interoception, by considering other pro-inflammatory markers and non-immunologic processes beyond neuroscience, such as childhood trauma [62], attachment styles [63], or stress regulation [64].

In the multivariate analysis, moderate evidence of a positive association between systemic low-grade inflammation and self-reported interoception was found for the MAIA-2 Not-Worrying scale, indicating reduced catastrophizing cognitions and emotional distress in response to pain or physical discomfort. The association was obscured in the bivariate analysis, suggesting a statistical suppression effect that was controlled for after inclusion of BMI. It may be counterintuitive that elevated CRP levels correlate with the tendency *not* to worry when experiencing pain or discomfort. For example, higher levels of CRP have been associated with lower pain thresholds and more physical pain, indicating altered pain processing in suicidal patients [65]. A meta-analysis also found decreased pain thresholds and lower tolerance for interoceptive noxious stimuli in individuals suffering from MDD compared to healthy controls [66]. However, consistent with our findings, Milaneschi et al. reported a protective effect of CRP on psychological symptom dimensions of depression, which was confirmed in a Mendelian randomization study linking genetically elevated CRP to a reduced risk of symptoms, such as suicidal ideation, feelings of inadequacy, anhedonia, cognitive problems, and anxiety [57]. Although statistical significance was not reached, the authors also found a CRP-predicted trend toward less worrying and better worry control [57]. Accordingly, the MAIA-2 Not-Worrying scale assesses psychological responses to pain and unpleasant bodily sensations rather than vulnerability to body sensations. Our findings contribute to a growing body of research that contradicts the notion that inflammatory activation is a general risk factor for mental health. For example, cumulative evidence suggests that higher serum levels of CRP protect individuals from developing schizophrenia, even though elevated CRP is correlated with disease activity after onset of schizophrenia [67,68]. With the recent advent of Mendelian randomization studies, further discrepancies have become apparent in depression research, where have challenges arisen in disentangling the protective effects of CRP from its risks [57].

The replication part of this study showed positive associations between serum levels of CRP and facets of multidimensional fatigue, which were in the expected directions [57]. Peripheral CRP specifically predicted both physical fatigue and reduced activity. These symptoms refer to the inflammatory phenotype of atypical depression, including physical exhaustion and leaden paralysis [12,14]. However, it has to be noted that recent findings from genetic Mendelian randomization studies suggest a causal involvement of IL-6 rather than CRP in the pathophysiology of fatigue [57,69]. Moreover, we found a positive association between peripheral CRP and overall severity of depression, which was consistent with meta-analytic evidence [70]. Congruent with past research, statistical adjustment for BMI in the multivariate analysis affected the strength of associations between the inflammatory marker and symptom dimensions of MDD [9,57,70], probably due to the confounding effect of obesity, which is moderately correlated with CRP [71].

The prevalence of overall inflammation (CRP > 3.0 mg/L: 37.12%) or acute inflammation (CRP > 10.0 mg/L: 8.25%) in our inpatient sample was consistent with findings from a meta-analysis reporting similar proportions [10]. These findings emphasized the significance of low-grade inflammation in a considerable subgroup of affected individuals who are at risk for treatment-resistant depression [15]. Elevated blood concentrations of CRP have been linked to subsequent risk of coronary heart disease, stroke, and vascular mortality [72]. However, there is still an ongoing debate about the source of inflammation in depression [73]. It has been suggested that MDD and chronic inflammatory conditions share common risk factors in their pathogenesis [31]. For example, a meta-analysis reported longitudinal associations between parental absence during early development and elevated CRP in adults suggesting a mechanism that may mediate the susceptibility to depression [74]. Additionally, an inflammatory mechanism has been proposed that links sleep disturbances to MDD [75]. 

From a methodological perspective, the replication of past research results supports the validity of our analysis, which applied modern Bayesian statistical methods. The Bayesian framework regularly faces major criticism considering the arbitrary selections of priors, which may reflect subjective assumptions of the researcher rather than objective criteria [53]. We faced these concerns by conducting a sensitivity analysis, which showed the robustness of our results, even after considering extreme priors. Fluctuations of the BFs were only marginal, without substantially influencing main findings. Against the background of the key findings of this study, we could demonstrate a major strength of the Bayesian statistical framework by quantifying evidence not only *against*, but also *for* the *H_0_*, which is not possible in conventional frequentist statistics, for epistemological reasons [41,45]. The application of Bayesian methods in future clinical research is strongly encouraged as computational power continues to increase.

The present study is subject to several limitations, as it is based on a cross-sectional analysis of secondary data, which precluded causal conclusions. The MAIA-2 Noticing, Not-Distracting, and Not-Worrying scales showed relatively low internal consistency reliability (*ω* < 0.70), which might have affected the validity of the results. Our findings may not be generalizable to proximal (e.g., IL-6, TNF-*α*) or other distal inflammatory markers. For example, certain symptoms of MDD are associated with a specific signature of inflammatory dysregulation [8]; a similar pattern can be observed in the long-term immunological sequelae of various types of childhood trauma [74]. Future research should therefore investigate the associations of other inflammatory mediators with facets of interoception and distinguish between central/peripheral inflammation. Our findings may also be replicated by including high-sensitivity CRP, which is more sensitive at lower concentrations [39]. Furthermore, we could not control for relevant confounding factors such as smoking status, alcohol consumption, cardiovascular fitness, and stress reactivity [54], because these variables were not available in the dataset. In addition, healthy controls were not recruited, limiting conclusions about the discriminative ability of CRP to predict dysfunctional vs. functional self-reported interoception. As we relied on sampling in an inpatient setting, the results of the study may not be generalizable to community samples with mild depression. Due to the limited sample size, we were unable to investigate sex as a potential moderator variable affecting the strength or direction of the associations examined, while sex-specific effects have been discussed for both CRP and interoception in MDD [9,28,76,77]. Our study was not sufficiently powered to derive conclusive evidence for all individual analyses. Future studies could use a sequential Bayes factor design that would allow researchers to stop collecting data once clear evidence *for* or *against* the *H_0_* was obtained [78].

## 5. Conclusions

Over a third of the included patients demonstrated low-grade or acute inflammation, as indicated by elevated CRP blood levels, but inflammatory responses were not associated with dysfunctional self-reported interoception. In contrast, systemic low-grade inflammation could potentially exert a protective effect against worries about unpleasant body sensations; a finding which merits future investigation. Preliminary evidence from our study suggests that anti-inflammatory treatment may not be appropriate to address deficits in self-reported interoception. However, an immunologic contribution to maladaptive interoception cannot be ruled out until future studies replicate our findings considering other inflammatory markers.

## Figures and Tables

**Table 1:** Sample characteristics stratified for CRP cut-points (*N* = 97).

Characteristics	Total	Peripheral CRP (mg/L)			
		<1.0	1.0–3.0	3.1–10.0	>10.0
*N* (Total %)	97 (100%)	8 (8.25%)	53 (54.64%)	28 (28.87%)	8 (8.25%)
Age (years, *M ± SD*)	47.56 ± 11.12	49.75 ± 11.50	47.55 ± 11.60	47.57 ± 10.21	45.38 ± 12.35
Female sex	53 (54.64%)	3 (37.50%)	30 (56.60%)	16 (57.14%)	4 (50.00%)
BMI (kg/m^2^, *M ± SD*)	26.31 ± 5.42	23.93 ± 4.70	24.90 ± 4.68	27.33 ± 4.40	34.45 ± 6.48
School Education					
≤9 years	23 (23.71%)	1 (12.50%)	13 (24.53%)	5 (17.86%)	4 (50.00%)
10 years	42 (43.30%)	5 (62.50%)	20 (37.74%)	14 (50.00%)	3 (37.50%)
≥11 years	32 (23.71%)	2 (25.00%)	20 (37.74%)	9 (32.14%)	1 (12.50%)
Vocational Education					
no vocational training	9 (9.28%)	0 (0.00%)	7 (13.21%)	2 (7.14%)	0 (0.00%)
vocational training	72 (74.23%)	7 (87.50%)	36 (67.92%)	22 (78.57%)	7 (87.50%)
academic degree	16 (16.49%)	1 (12.50%)	10 (18.87%)	4 (14.29%)	1 (12.50%)
Employment status					
unemployed	20 (20.62%)	2 (25.00%)	12 (22.64%)	4 (14.29%)	2 (25.00%)
employed	66 (68.04%)	4 (50.00%)	36 (67.92%)	22 (78.57%)	4 (50.00%)
retired	11 (11.34%)	2 (25.00%)	5 (9.43%)	2 (7.14%)	2 (25.00%)
Main diagnosis (ICD-10)					
Single depr. episode (F32)	29 (29.90%)	3 (37.50%)	17 (32.08%)	8 (28.57%)	1 (12.50%)
Recurrent depr. disorder (F33)	68 (70.10%)	5 (62.50%)	36 (67.92%)	20 (71.43%)	7 (87.50%)
Severity of depression (ICD-10)					
Moderate (F3x.1)	12 (12.37%)	1 (12.50%)	6 (11.32%)	3 (10.71%)	2 (25.00%)
Severe without psychotic features (F3x.2)	85 (87.63%)	7 (87.50%)	47 (88.68%)	25 (89.29%)	6 (75.00%)
Number of past psychiatric inpatient stays (self-report, *M ± SD*)	1.39 ± 1.78	0.75 ± 0.71	1.47 ± 1.68	1.46 ± 2.15	1.25 ± 1.83
Somatic comorbidity (yes)	30 (30.93%)	0 (0.00%)	13 (24.53%)	12 (42.86%)	5 (62.50%)
Medication					
Psychotropic drugs at admission (self-reported number, *M ± SD*)	1.40 ± 1.26	1.38 ± 1.06	1.36 ± 1.19	1.39 ± 1.34	1.75 ± 1.75
Statins (yes)	9 (9.28%)	1 (12.50%)	4 (7.55%)	3 (10.71%)	1 (12.50%)
Antihypertensives (yes)	27 (27.84%)	2 (25.00%)	12 (22.64%)	8 (28.57%)	5 (62.50%)
MAIA-2					
Noticing (*M ± SD*)	2.98 ± 1.02	2.97 ± 0.97	2.96 ± 1.19	3.08 ± 0.75	2.75 ± 0.83
Not-Distracting (*M ± SD*)	1.81 ± 0.81	1.77 ± 0.90	1.73 ± 0.82	1.92 ± 0.77	1.98 ± 0.88
Not-Worrying (*M ± SD*)	2.01 ± 0.94	2.25 ± 0.50	1.98 ± 1.01	2.12 ± 0.85	1.52 ± 0.97
Attention Regulation (*M ± SD*)	2.04 ± 0.92	2.36 ± 0.88	2.04 ± 0.89	1.95 ± 1.00	2.00 ± 0.94
Emotional Awareness (*M ± SD*)	3.31 ± 1.15	3.28 ± 0.98	3.25 ± 1.28	3.40 ± 0.86	3.38 ± 1.49
Self-Regulation (*M ± SD*)	1.64 ± 0.91	1.41 ± 0.48	1.67 ± 0.90	1.64 ± 0.92	1.72 ± 1.35
Body Listening (*M ± SD*)	1.53 ± 1.02	2.29 ± 0.55	2.12 ± 1.26	2.20 ± 1.28	1.92 ± 1.22
Trusting (*M ± SD*)	2.14 ± 1.21	2.29 ± 0.55	2.12 ± 1.26	2.20 ± 1.28	1.92 ± 1.22
BDI-II (*M ± SD*)	31.32 ± 10.29	23.00 ± 9.50	31.96 ± 10.93	30.96 ± 8.16	36.62 ± 10.20
MFI-20					
General Fatigue (*M ± SD*)	15.88 ± 3.34	13.50 ± 3.51	16.06 ± 3.25	15.75 ± 3.49	17.50 ± 2.33
Physical Fatigue (*M ± SD*)	14.72 ± 3.87	10.88 ± 2.75	14.83 ± 3.88	14.71 ± 3.63	17.88 ± 2.53
Mental Fatigue (*M ± SD*)	15.67 ± 3.26	13.00 ± 4.96	15.83 ± 3.08	15.79 ± 2.97	16.88 ± 2.53
Reduced Activity (*M ± SD*)	15.09 ± 3.77	12.75 ± 3.92	15.11 ± 3.50	15.36 ± 4.35	16.38 ± 2.72
Reduced Motivation (*M ± SD*)	14.03 ± 3.43	11.88 ± 3.60	14.42 ± 3.07	13.57 ± 3.99	15.25 ± 2.92

Note: *M ± SD* = mean ± standard deviation; *N* = absolute frequency; % = relative frequency; BMI = body mass index; BDI-II = Beck Depression Inventory-II; ICD-10 = International Statistical Classification of Diseases and Related Health Problems (10th revision); MFI-20 = Multidimensional Fatigue Inventory; MAIA-2 = Multidimensional Assessment of Interoceptive Awareness, Version 2; CRP = C-reactive protein.

**Table 2:** Bayesian zero-order correlations and sensitivity analysis (*N* = 97).

Scale	Bayesian Correlation with log_10_ CRP			Sensitivity Analysis with Varying Priors		
	*r_Median_*	95% HDI	BF_10_ (*γ*_1_ = 1/3)	BF_10_ ($\gamma_{2}=1/\sqrt{27}$)	BF_10_ ($\gamma_{3}=1/\sqrt{3}$)	BF_10_ (*γ*_4_ = 1)
MAIA-2						
Noticing ^1^	0.00	[−0.17, 0.21]	0.23	0.31	0.17	0.13
Not-Distracting ^1^	0.11	[−0.08, 0.29]	0.43	0.55	0.33	0.24
Not-Worrying ^1^	−0.03	[−0.20, 0.17]	0.24	0.32	0.18	0.13
Attention Regulation ^1^	−0.08	[−0.26, 0.11]	0.32	0.42	0.24	0.18
Emotional Awareness ^1^	0.02	[−0.16, 0.22]	0.24	0.31	0.18	0.13
Self-Regulation ^1^	0.02	[−0.15, 0.23]	0.24	0.31	0.18	0.13
Body Listening ^1^	0.02	[−0.16, 0.22]	0.24	0.31	0.18	0.13
Trusting ^1^	−0.01	[−0.18, 0.19]	0.23	0.31	0.18	0.13
BDI-II ^2^	0.21	[0.03, 0.39]	3.19	3.86	2.51	1.89
MFI-20						
General Fatigue ^2^	0.16	[0.01, 0.34]	1.22	1.52	0.97	0.70
Physical Fatigue ^2^	0.28	[0.09, 0.46]	20.64	23.14	16.98	13.11
Mental Fatigue ^2^	0.15	[0.01, 0.33]	1.01	1.27	0.77	0.57
Reduced Activity ^2^	0.22	[0.04, 0.40]	4.67	5.55	3.70	2.79
Reduced Motivation ^2^	0.14	[0.01, 0.32]	0.80	1.01	0.61	0.45

Note: BDI-II = Beck Depression Inventory-II; MFI-20 = Multidimensional Fatigue Inventory; MAIA-2 = Multidimensional Assessment of Interoceptive Awareness, Version 2; log_10_ CRP = log-normalized C-reactive protein (CRP); *r_Median_* = Median Bayesian correlation derived from the posterior distribution (robust measure of centrality); 95% HDI = 95% highest density interval (credible interval); BF_10_ = Bayes Factor (H_1_ (nominator) against H_0_ (denominator)); γ = scaling factor for the shifted beta distribution (simulated with the R ‘correlationBF’ function and 10,000 iterations). ^1^ two-sided test (H_0_: *ρ* = 0; H_1A_: *ρ* ≠ 0). ^2^ one-sided test (H_0_: *ρ* = 0; H_1B_: *ρ* > 0).

**Table 3:** Adjusted associations of CRP with multidimensional self-reported interoception, depression, and fatigue severity (*N* = 97).

Scale	Predictor: log_10_ CRP (Overall Inflammation, *N* = 97)				Sensitivity Analysis (Exclusion of Acute Inflammation, *N* = 89)			
	*β_Median_* [95% HDI]	*b_Median_* [95% HDI]	ESS	BF	*β_Median_* [95% HDI]	*b_Median_* [95% HDI]	ESS	BF
MAIA-2								
Noticing ^1^	−0.08 [−0.33, 0.18]	−0.21 [−0.90, 0.48]	21,421	0.59	0.01 [−0.22, 0.26]	0.05 [−0.75, 0.86]	24,469	0.56
Not-Distracting ^1^	0.15 [−0.10, 0.41]	0.33 [−0.22, 0.87]	21,130	0.73	0.20 [−0.03, 0.43]	0.52 [−0.09, 1.12]	23,606	1.25
Not-Worrying ^1^	0.26 [0.03, 0.50]	0.66 [0.06, 1.24]	20,833	2.69	0.28 [0.06, 0.52]	0.84 [0.15, 1.51]	23,395	3.97
Attention Regulation ^1^	−0.05 [−0.29, 0.20]	−0. 11 [−0.71, 0.49]	24,267	0.34	−0.04 [−0.27, 0.19]	−0.13 [−0.81, 0.55]	22,453	0.39
Emotional Awareness ^1^	−0.03 [−0.28, 0.24]	−0.08 [−0.88, 0.71]	21,465	0.35	0.01 [−0.24, 0.25]	0.02 [−0.88, 0.90]	22,697	0.41
Self-Regulation ^1^	−0.02 [−0.29, 0.22]	−0.06 [−0.69, 0.55]	21,710	0.34	0.02 [−0.22, 0.26]	0.06 [−0.62, 0.74]	23,575	0.37
Body Listening ^1^	−0.02 [−0.27, 0.24]	−0.04 [−0.73, 0.66]	22,118	0.33	0.01 [−0.23, 0.25]	0.02 [−0.77, 0.79]	22,465	0.40
Trusting ^1^	0.00 [−0.26, 0.25]	0.00 [−0.82, 0.82]	22,109	0.33	0.00 [−0.25, 0.25]	0.00 [−0.95, 0.96]	19,393	0.35
BDI-II ^2^	0.24 [−0.01, 0.48]	6.33 [−0.37, 13.08]	22,177	1.43	0.13 [−0.09, 0.36]	4.17 [−3.24, 11.63]	23,479	0.66
MFI-20								
General Fatigue ^2^	0.08 [−0.16, 0.33]	0.74 [−1.46, 2.91]	21,148	0.35	0.02 [−0.21, 0.27]	0.27 [−2.37, 2.89]	23,136	0.36
Physical Fatigue ^2^	0.24 [−0.01, 0.48]	2.44 [−0.07, 4.95]	22,672	6.11	0.13 [−0.12, 0.37]	1.56 [−1.43, 4.52]	22,856	0.55
Mental Fatigue ^2^	0.23 [−0.03, 0.47]	1.98 [−0.23, 4.14]	21,937	1.27	0.14 [−0.10, 0.39]	1.53 [−1.09, 4.16]	21,201	0.63
Reduced Activity ^2^	0.34 [0.09, 0.57]	3.38 [0.94, 5.76]	21,939	7.35	0.29 [0.05, 0.52]	3.53 [0.63, 6.46]	23,116	3.24
Reduced Motivation ^2^	0.19 [−0.07, 0.44]	1.71 [−0.59, 4.09]	22,722	0.79	0.14 [−0.10, 0.39]	1.54 [−1.18, 4.26]	23,865	0.59

Note: BDI-II = Beck Depression Inventory-II; MFI-20 = Multidimensional Fatigue Inventory; MAIA-2 = Multidimensional Assessment of Interoceptive Awareness, Version 2; *β_Median_*/*b_Median_* = unstandardized/standardized regression coefficient (robust median of the posterior distribution); BF = likelihood of the full model against the reduced model omitting log_10_ CRP (= individual contribution of log_10_ CRP to the model); 95% HDI = 95% highest density interval (credible interval); ESS = effective sample size; log_10_ CRP = log-normalized C-reactive protein (CRP). ^1^ The models were adjusted for age, sex, BMI, school education, employment status, somatic comorbidity, depression severity, intake of antihypertensives, and statins. ^2^ The models were adjusted for age, sex, BMI, school education, employment status, somatic comorbidity, intake of antihypertensives, and statins.

## Data Availability

The datasets used and/or analyzed during the current study are available from the corresponding author on reasonable request.
